# *CaPHOT1* Negatively Regulates the Pepper Resistance to *Phytophthora capsici* Infection

**DOI:** 10.3390/plants14213400

**Published:** 2025-11-06

**Authors:** Ying Luo, Hongyan Liu, Huiling Zhu, Feng Yang, Yanli Tu, Ting Yu, Yong Zhou, Youxin Yang

**Affiliations:** 1 Jiangxi Provincial Key Laboratory for Postharvest Storage and Preservation of Fruits & Vegetables, College of Agronomy, Jiangxi Agricultural University, Nanchang 330045, China; luoying1934@163.com (Y.L.); liuhongyan9695@163.com (H.L.); zhuhuiling0201@163.com (H.Z.); yuting@jxau.edu.cn (T.Y.); 2 Key Laboratory of Crop Physiology, Ecology and Genetic Breeding, Ministry of Education, College of Bioscience and Bioengineering, Jiangxi Agricultural University, Nanchang 330045, China; yf635343@163.com; 3 Nanchang Agricultural Technology Extension Center, Nanchang 330038, China; tuyanli2025@163.com

**Keywords:** pepper, phototropin (PHOT), *Phytophthora capsici*, defense resistance, gene expression

## Abstract

Phototropins (PHOTs) are plant blue-light receptors that mediate crucial physiological processes such as phototropism, chloroplast movement, stomatal opening, and flowering. However, the *PHOT* family genes remain poorly characterized in pepper. Here, we identified and molecularly cloned two *PHOT* genes (*CaPHOT1* and *CaPHOT2*) in pepper, which were phylogenetically classified into distinct groups with their homologs from rice, maize, tomato, and *Arabidopsis*. These genes exhibit conserved gene structures, implying functional conservation during evolution. Subcellular localization analysis confirmed that both CaPHOT1 and CaPHOT2 are localized to the plasma membrane. Expression profiling revealed that both *CaPHOT1* and *CaPHOT2* were expressed in all tissues, with the highest transcripts in leaves and the lowest in roots. Notably, RNA-seq data revealed that the expression of *CaPHOT1* was up-regulated by JA and SA, whereas *CaPHOT2* showed no significant changes. Furthermore, *CaPHOT1* and *CaPHOT2* displayed divergent expression patterns upon *Phytophthora capsici* infection (PCI). Furthermore, transient overexpression of *CaPHOT1* in pepper enhanced susceptibility to PCI, indicating its negative role in disease resistance. Our findings identified the *CaPHOT* gene family in pepper and functionally demonstrated that *CaPHOT1* negatively regulates resistance to PCI, thereby providing insights for future research on *PHOTs* in other plant species.

## 1. Introduction

Light plays a crucial role in plant growth and development, serving not only as the energy source for photosynthesis but also as an environmental signal regulating plant growth [1]. Plants possess a sophisticated light-responsive system composed of a series of photoreceptor proteins, which perceive light radiation information (such as light intensity, quality, direction, and photoperiod) and transduce light signals to regulate various biological processes. Most plants possess five types of specialized photoreceptors. Amongst them, blue light orchestrates a wide spectrum of plant developmental processes through the coordinated action of three principal classes of blue-light receptors. These receptors include phototropin (PHOT), cryptochrome (CRY), and ZTL/FKF1/LKP2 (ZEITLUPE/FLAVIN-BINDER KELCH REPEAT F-BOX 1/LOV KELCH PROTEIN 2), which play distinct yet interconnected roles in mediating photomorphogenic responses [2,3]. CRYs mediate de-etiolation, shade avoidance, floral induction, and responses to abiotic and biotic stresses [4,5], while the ZTL/FKF1/LKP2 proteins are involved in architecture development [6], chloroplast movement [7], circadian clock and photoperiodic flowering [8,9,10].

As one of the blue-light receptors, PHOT is involved in the perception and transduction of blue light signal and primarily regulate plant movement responses, including phototropism, chloroplast movement, stomatal opening, leaf positioning and flattening [11,12]. Previously, two PHOT proteins were identified in *Arabidopsis* and designated as PHOT1 and PHOT2 [13]. They can perceive blue light and differentially activated, which establishes an auxin gradient that drives phototropic growth [14]. This process is dependent on the essential kinase activity of PHOTs and the phosphorylation of residues within the activation loop of their kinase domains. Subsequent studies have also identified PHOT family members in various plant species, such as rice [15], maize [16], *Phalaenopsis aphrodite* [17], cotton [18], and *Brassica* species [19]. Structural analysis reveals that PHOTs are AGC (cAMP-dependent protein kinase A, cGMP-dependent protein kinase G, and phospholipid-dependent protein kinase C) family kinases with molecular weights of approximately 120 kDa. Their N-terminal region contains two light-sensing light oxygen voltage (LOV) domains (LOV1 and LOV2) that bind the blue light-absorbing chromophore flavin mononucleotide (FMN) [20,21], while the C-terminal region consists of a serine/threonine kinase (STK) domain characteristic of the AGC kinase family. Following blue light stimulation, PHOTs undergo autophosphorylation, which modulates their kinase activity and subsequently phosphorylate their substrate proteins, thereby regulating a range of physiological responses [22]. The phosphorylation dynamics of PHOT1 can be regulated by HDA9-mediated deacetylation and ultimately govern the phototropic response in plants [23].

Studies across diverse plant species have demonstrated that PHOTs mediate movement responses in a blue light dependent manner, such as hypocotyl elongation, phototropism, chloroplast movement, and stomatal opening. For example, constitutive expression of *GhPHOT2-1* restored the chloroplast avoidance response in the *Arabidopsis phot2* mutant, while its silencing disrupted this movement in cotton, demonstrating that *GhPHOT2-1* is a mediator of chloroplast avoidance response [18]. Besides plant movement responses, some *PHOT* genes have been found to participate in other physiological processes in plants. For example, strawberry FaPHOT2 plays a positive role in blue light-mediated changes in anthocyanin biosynthesis and accumulation [24]. Besides functioning redundantly with SlPHOT2 in chloroplast relocation, tomato SlPHOT1 also plays a critical role in regulating carotenoid biosynthesis during fruit ripening [25]. Stphot1 negatively regulates plant immunity to *P. infestans* by activating StNRL1 through its blue-light-induced kinase activity, which subsequently targets the immune positive regulator StSWAP70 for proteasomal degradation [26]. A recent report revealed that PHOT2 integrates blue light and low-temperature signals with CAMTA2 to regulate flowering time in *Arabidopsis* through the NPH3-mediated signaling pathway [27].

Pepper (*Capsicum* spp.) is a globally important and profitable horticultural crop whose productivity is significantly constrained by various biotic stresses. Among them, *Phytophthora capsici* can cause a devastating disease, severely stunting plant growth and compromising fruit quality, which leads to substantial economic losses. Although some genes involved in pepper disease resistance have been identified, the genetic basis of PCI resistance remains incompletely understood. Therefore, it is imperative to explore and characterize additional genetic resources in pepper that contribute to the regulation of defense responses against PCI. In this study, two *CaPHOT* genes (*CaPHOT1* and *CaPHOT2*) were identified and cloned in pepper. A comprehensive analysis was subsequently conducted to characterize their phylogenetic relationships, gene structures, and expression patterns across various tissues and in response to jasmonic acid (JA), salicylic acid (SA), and *Phytophthora capsici* infection (PCI). Notably, *CaPHOT1* was significantly responded to JA, SA and PCI. Subsequently, transient overexpression in pepper preliminarily confirmed the negative role of *CaPHOT1* during PCI. Our findings lay the foundation for in-depth analysis of *CaPHOT1* function in blue light-mediated pepper immunity against PCI and opens new avenues for research on *PHOT* gene functions.

## 2. Materials and Methods

### 2.1. Identification and Cloning of PHOT Genes in Pepper

The amino acid sequences of *Arabidopsis thaliana* PHOT proteins were retrieved from the TAIR database (http://www.arabidopsis.org/) (accessed on 3 October 2025) based on a previous study [13]. A BLASTP search was performed against the online Sol Genomics Network (SGN) database (https://www.solgenomics.net/) (accessed on 3 October 2025) using the AtPHOT protein sequences as queries to identify homologs in pepper. From the SGN database, the sequence information for *CaPHOTs*, including their coding sequence (CDS), genomic sequence, and encoded amino acid sequence, was downloaded. The physicochemical properties of the CaPHOT proteins were analyzed using the ProtParam tool (https://web.expasy.org/protparam/) (accessed on 3 October 2025). The subcellular localization of CaPHOT proteins was predicted using the ProtComp 9.0 server (http://www.softberry.com/) (accessed on 3 October 2025).

### 2.2. Multiple Sequence Alignment, Phylogenetic and Gene Structure Analysis

For sequence alignment of the pepper PHOT proteins, the full-length protein sequences from pepper, rice [15], maize [16], tomato [28], and *Arabidopsis* were aligned using MEGA 7.0 software with the default parameters. The resulting multiple sequence alignment was then visualized using the GeneDoc software. Furthermore, a phylogenetic tree was constructed from this alignment with MEGA 7.0 by applying the neighbor-joining (NJ) method using 1000 replicates of bootstrap analysis, with the other parameters set as default. Additionally, the gene structure diagrams of *PHOT* genes, illustrating the distribution of exons and introns, were generated and analyzed with the GSDS tool (http://gsds.gao-lab.org/) (accessed on 3 October 2025) [29], by comparing the CDS and corresponding genomic DNA (gDNA) sequences.

### 2.3. Analysis of RNA-Seq Data of CaPHOT Genes

To analyze the expression of pepper *PHOT* genes in response to jasmonic acid (JA) and salicylic acid (SA) treatment, RNA-seq data were obtained from the PepperHub (http://lifenglab.hzau.edu.cn/PepperHub/index.php) (accessed on 3 October 2025) [30]. The dataset included leaf samples collected at seven time points (0, 0.5, 1, 3, 6, 12, and 24 h) following JA and SA treatments. The expression levels of *CaPHOT* genes were quantified and are presented as fragments per kilobase per million reads (FPKM) values, following the methodology of a previous study [31].

### 2.4. Plant Materials and Pathogen Preparation

Seeds of pepper cultivar ‘No. 8’ were soaked in warm water for 4 h and then germinated in an incubator at 28 °C. After radicle emergence, the seeds were transferred to seedling trays in a growth chamber with a 12 h photoperiod, day/night temperatures of 25/20 °C, and relative humidity of 70%. Various tissues, including root, stem, flower, fruit, leaf, and placenta, were collected from normally growing pepper plants. Pepper plants at the six-true-leaf stage were inoculated with *Phytophthora capsici*, and leaf samples were collected from control (0 h) plants and at 12, 24, 48, 72 and 96 h post-inoculation. All collected samples were immediately frozen in liquid nitrogen and stored at −80 °C for subsequent expression analysis. Three independent biological replicates were set up for the experiment.

Pathogen preparation was performed following the method described in our previous study [32]. Briefly, the *P. capsici* strain “JX1” was cultured on 10% V8 medium (containing 100 mL V8 juice, 1 g CaCO_3_, and 15 g agar per liter) at 28 °C in the dark for 10 days to induce sporangia production. On the evening prior to inoculation, the cultures were washed three times with sterile water at 30 min intervals. The following day, 10 mL of sterile water was added to each dish, which were then incubated at 4 °C for 40 min, followed by 28 °C for 15 min until zoospores were released, and the zoospore concentration was determined using a hemocytometer and adjusted to 1 × 10^5^ spores/mL.

### 2.5. Transient Overexpression of CaPHOT1 in Pepper

The coding region of the *CaPHOT1* were amplified and inserted into the PAC overexpression vector, and the resulting recombinant vector was designated as PAC-CaPHOT1. *Agrobacterium tumefaciens* GV3101 strains harboring either the PAC-CaPHOT1 construct or the empty PAC vector (control) were inoculated into liquid LB medium and cultured overnight at 28 °C. Finally, the bacteria were resuspended in infiltration buffer (10 mM MgCl_2_, 10 mM MES, 100 μM acetosyringone, pH 5.6), and the concentration was adjusted to an OD_600_ of 0.8 using a spectrophotometer. When pepper plants reached the 6–8 leaf stage under the aforementioned growth conditions, the *Agrobacterium* suspension was drawn into a sterile needle-free disposable syringe. Twenty pepper leaves of similar size and developmental stage were randomly selected from plants at the 6–8 leaf stage grown under the aforementioned conditions for whole-leaf infiltration. After infiltration, the plants were maintained in a growth chamber at 25 °C under a 16 h light/8 h dark photoperiod. At 36 h post-infiltration, the infiltrated leaves were excised and placed on agar medium. A 20 μL droplet of a zoospore suspension (about 2000 spores) was inoculated onto each leaf. At 3 days post-inoculation, lesion sizes were measured and photographed under UV light, as well as stained by simmering in trypan blue solution on the basis of the previously described method [32].

### 2.6. Subcellular Localization of CaPHOT Proteins

The coding regions of the *CaPHOT1* and *CaPHOT2* (without the stop codon) were amplified and then ligated into pSuper1300-GFP vector using the ClonExpress^®^ II One Step Cloning Kit (Vazyme, Nanjing, China). The recombinant plasmids were transformed into *E. coli*, and positive clones were screened by PCR. Plasmids from positive clones were sent for sequencing, and the successfully recombinant plasmids were obtained. The recombinant plasmid was subsequently transformed into *Agrobacterium tumefaciens* GV3101. The resuspended bacterial solution was injected into the abaxial side of leaves of 4–6-week-old *Nicotiana benthamiana* plants using agrobacterium-mediated transformation as previously described [33]. For the experimental group, the CaPHOT-GFP construct was co-transformed with pm-rk, a red fluorescent plasma membrane marker [34], into N. *benthamiana* leaf epidermal cells. For the control group, the empty pSuper1300-GFP vector was co-injected with the RFP plasma membrane marker pm-rk. After incubating the plants for 2–3 days, protein localization was observed using confocal laser scanning microscopy (Nikon Corporation, Tokyo, Japan).

### 2.7. RNA Isolation and Quantitative RT-PCR (qRT-PCR)

Total RNA was extracted with the TRIzol method using TRIpure Reagent (Wuhan Keep Biotechnology, Wuhan, China), and first-strand cDNA was synthesized using Hifair^®^ III 1st Strand cDNA Synthesis Kit (gDNA digester plus) (Yeasen, Shanghai, China) according to the manufacturer’s instructions. Expression analysis of *CaPHOT* genes was performed in three replicates by quantitative real-time PCR (qRT-PCR) with pepper actin (*CaActin*) as the internal reference as previously described [33]. The reaction program consisted of an initial step at 95 °C for 5 min, followed by 40 cycles of 95 °C for 10 s and 60 °C for 30 s. Their relative transcript levels were analyzed by using the 2^−ΔΔCt^ method [35]. The gene-specific primers used for qRT-PCR are provided in Table S1.

### 2.8. Statistical Analysis

All statistical analyses were performed using SPSS Statistics 20.0 (SPSS Inc., Chicago, IL, USA), with graphical presentations generated using GraphPad Prism 9.5. All values are represented as mean ± SDs of three biological replicates. Statistical significance was determined by one-way ANOVA followed by Tukey’s multiple comparison test.

## 3. Results

### 3.1. Cloning and Identification of PHOT Genes of Pepper

To identify the *PHOT* genes in the pepper genome, a BLASTp search was performed using *Arabidopsis* PHOT protein sequences as queries to search for their homologs in the SGN database. Two pepper *PHOT* genes were identified and designated as *CaPHOT1* and *CaPHOT2* based on their homology to *Arabidopsis* genes (Table 1). To verify the accuracy of the sequences obtained from the pepper genome, specific primers were designed according to the CDSs of *CaPHOT1* and *CaPHOT2*. Using cDNA from pepper leaf tissues as a template, the 3078 bp and 2883 bp CDSs of *CaPHOT1* and *CaPHOT2* were successfully amplified (Figure S1). Sequencing of the PCR products confirmed the CDSs of both genes, supporting the reliability of the identified sequences. Table 1 summarizes the physicochemical properties of *CaPHOT1* and *CaPHOT2*. The genomic DNA lengths of *CaPHOT1* and *CaPHOT2* were 18,295 bp and 12,575 bp, while their CDS lengths were 3078 bp and 2883 bp, encoding proteins of 1025 and 960 amino acids, respectively (Table 1). The molecular weight (MW) of CaPHOT1 and CaPHOT2 were predicted to be 115.39 kDa and 107.20 kDa, with isoelectric point (pI) of 6.48 and 8.09, and grand averages of hydropathicity (GRAVY) of −0.706 and −0.538, respectively. Subcellular localization analysis using ProtComp 9.0 predicted that both CaPHOT proteins are localized to the plasma membrane (Table 1).

### 3.2. Phylogenetic and Gene Structure Analyses of the PHOT Gene Family Among Different Plant Species

**Figure 1:**
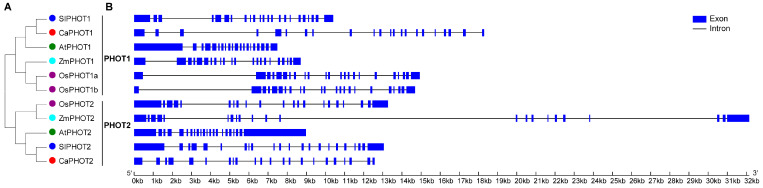
Phylogenetic and gene structure of PHOT members from different plant species. (**A**) Phylogenetic tree of PHOT protein sequences. (**B**) Exon–intron structure of *PHOT* genes.

To elucidate the phylogenetic relationships of PHOT proteins, a phylogenetic tree was constructed using the full-length PHOT protein sequences from pepper, rice, maize, tomato, and *Arabidopsis*. The results indicate that PHOT family members from these species were divided into two distinct groups, designated PHOT1 and PHOT2 (Figure 1A). Additionally, the pepper PHOT members showed the closest genetic relationship to those of tomato and *Arabidopsis* than rice and maize, consistent with two well-supported clades (PHOT1/PHOT2) across sampled dicots/monocots.

To further understand the evolution of PHOT members, we compared the exon–intron structures of *PHOT* genes from the above five plant species. As a result, *PHOT* genes from PHOT1 group contained 19–22 introns, while *PHOT* genes from PHOT2 group had 20–21 introns (Figure 1B). Most of the *PHOT* genes (including *CaPHOT1* and *CaPHOT2*) possessed 21 introns, two *OsPHOT* genes (*OsPHOT1a* and *OsPHOT1b*) contained 22 introns, whereas *AtPHOT1* had 19 introns (Figure 1B).

### 3.3. Conserved Domain Analysis of CaPHOT Proteins

To understand the conserved domain features of CaPHOT proteins, multiple sequence alignments of PHOT protein sequences from pepper, rice, maize, tomato, and *Arabidopsis* were also performed. The N-terminus of these PHOT proteins consists of two similar LOV1 and LOV2 domains, while a serine/threonine kinase (STK) domain is located at the C-terminus (Figure S2).

### 3.4. Expression Profiles of CaPHOT Genes in Various Tissues

**Figure 2:**
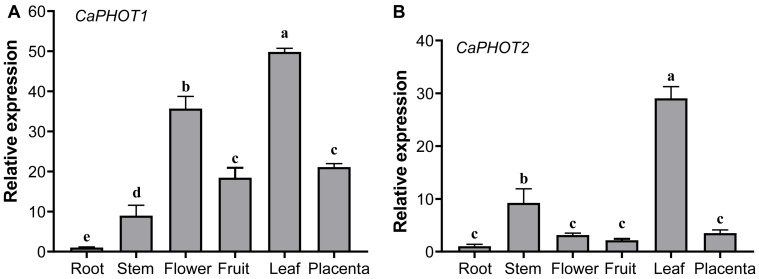
Tissue-specific expression analysis of *CaPHOT1* (**A**) and *CaPHOT2* (**B**) in pepper. Expression levels were determined by qRT-PCR with three biological replicates and normalized to the value in the root tissue (set as 1.0). Statistical significance was assessed by one-way ANOVA with Tukey’s test, and different lowercase letters indicate significant differences (*p* < 0.05).

The expression of *CaPHOT1* and *CaPHOT2* in root, stem, flower, fruit, leaf, and placenta was analyzed using qRT-PCR. The results showed that both *CaPHOT1* and *CaPHOT2* are expressed in various tissues, with the highest expression levels in leaf and the lowest in root (Figure 2). In addition, *CaPHOT1* also exhibits relatively high expression in flower (Figure 2A), while *CaPHOT2* shows relatively high expression in stem (Figure 2B).

### 3.5. Expression of CaPHOT Genes Under JA and SA Treatments

**Figure 3:**
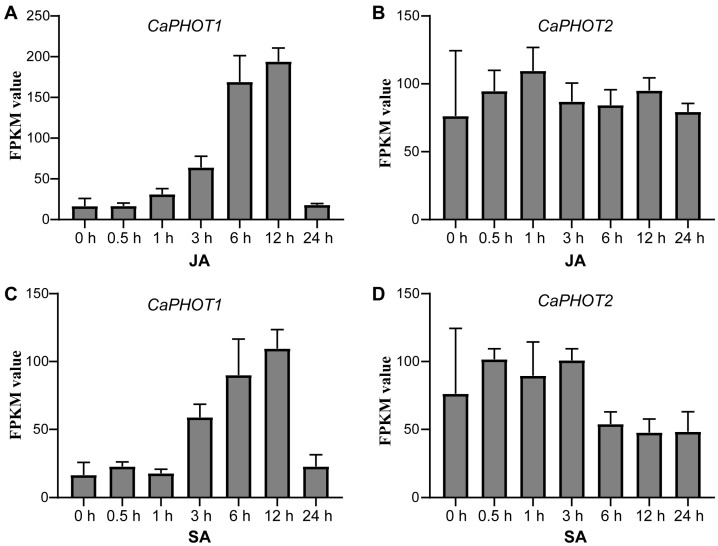
Expression analysis of *CaPHOT1* and *CaPHOT2* in response to JA and SA treatments. (**A**,**B**) Expression of *CaPHOT1* (**A**) and *CaPHOT2* (**B**) under JA treatment at different time points according to the RNA-seq data. (**C**,**D**) Expression of *CaPHOT1* (**C**) and *CaPHOT2* (**D**) under SA treatment at different time points according to the RNA-seq data.

To gain insights into the possible functions of *CaPHOT* genes under JA and SA treatments, we analyzed their expression profiles using publicly available RNA-seq data [30]. Under JA treatment, the expression of *CaPHOT1* was up-regulated until 12 h but decreased sharply at 24 h (Figure 3A). In contrast, the expression of *CaPHOT2* showed no significant change (Figure 3B). Under SA treatment, *CaPHOT1* exhibited a comparable pattern to that under JA treatment, with a gradual increase peaking at 12 h and subsequent down-regulation at 24 h (Figure 3C). Likewise, no significant alteration was detected in the expression of *CaPHOT2* under SA treatment (Figure 3D).

### 3.6. Expression Patterns of CaPHOT Genes Under P. capsici Infection

**Figure 4:**
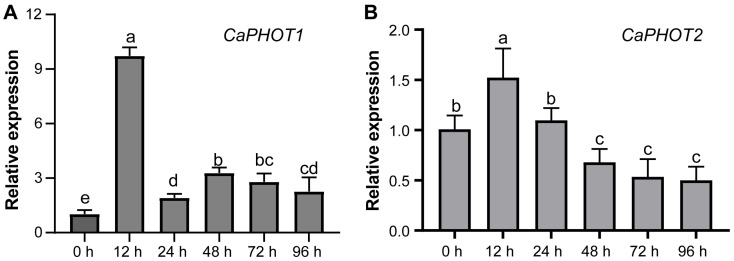
qRT-PCR analyses of levels of CaPHOT1 (**A**) and CaPHOT2 (**B**) transcripts under PCI at different time points. Data (mean ± SD) represent three replicates analyzed using the 2^−ΔΔCt^ method, with the expression level of each *CaPHOT* gene at 0 h set as “1.0”. Statistical significance was assessed by one-way ANOVA with Tukey’s test, and different letters above the columns indicate significant differences (*p* < 0.05).

To investigate the potential role of *CaPHOT* genes in pepper’s defense against *P. capsici* infection, we analyzed their expression levels at different time points under PCI using qRT-PCR. The results showed that the expression of both *CaPHOT1* and *CaPHOT2* exhibited an initial increase and peaking at 12 h, followed by a decrease at the later time points (Figure 4). This expression pattern suggests that *CaPHOT* genes may be functionally involved in defense response against PCI in pepper.

### 3.7. Subcellular Localization of CaPHOT1 and CaPHOT2

**Figure 5:**
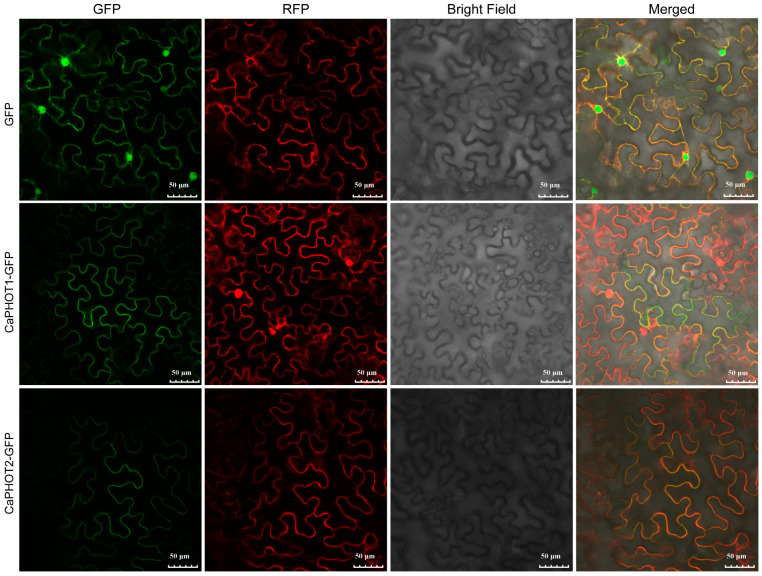
Subcellular localization of CaPHOT1 and CaPHOT2. The green fluorescent protein (GFP) fluorescence, red colored pm-rk plasma membrane marker, bright field, and the merged images are shown. Scale bar = 50 μm.

To assess the subcellular localization of CaPHOT1 and CaPHOT2, their full-length coding sequences (without stop codons) were fused in-frame to the N-terminus of green fluorescent protein (GFP) under the control of the cauliflower mosaic virus 35S promoter. These fusion constructs were transiently co-expressed with a plasma membrane marker in *Nicotiana benthamiana* leaves. Confocal microscopy revealed that the GFP fluorescence signal co-localized with the plasma membrane marker (Figure 5), indicating that both CaPHOT1 and CaPHOT2 are localized to the plasma membrane.

### 3.8. Overexpression of CaPHOT1 in Pepper Reduces the Resistance to P. capsici Infection

**Figure 6:**
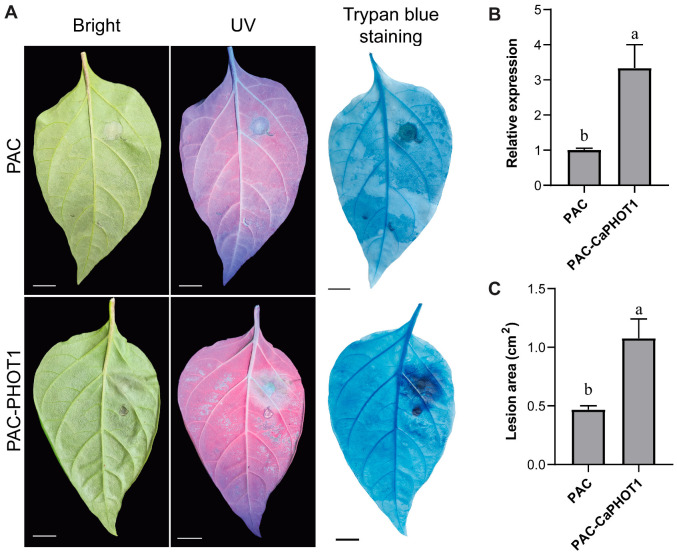
Transient overexpression of CaPHOT1 reduces pepper resistance to PCI. (**A**) Phenotypes of de-tached leaves of PAC and PAC-CaPHOT1 pepper plants after P. *capsici* infection. (**B**) Expression analysis of CaPHOT1 in leaves of PAC and PAC-CaPHOT1 pepper plants using qRT-PCR with three biological replicates. RNA was extracted from the leaf tissues adjacent to the inoculation sites. (**C**) Lesion area determination of detached leaves of PAC and PAC-CaPHOT1 pepper plants after inoculation with *P. capsici*. Statistical significance was assessed by one-way ANOVA with Tukey’s test, and different letters stand for significant differences between groups at *p* < 0.05.

We selected CaPHOT1 for subsequent functional analysis because its transcriptional response to PCI was significantly more pronounced than that of *CaPHOT2* (Figure 4). To further analyze the role of *CaPHOT1* in the pepper defense response to PCI, an overexpression vector (PAC-CaPHOT1) was constructed. Its effect was examined using an *Agrobacterium*-mediated transient expression assay in pepper leaves according to our previous study [33]. The results showed that transient expression of *CaPHOT1* induced a larger water-soaked lesion area and darker trypan blue staining compared to the control (Figure 6), indicating that overexpression of *CaPHOT1* reduces pepper resistance to PCI.

## 4. Discussion

In recent years, increasing studies have focused on plant responses to blue light and the underlying signal transduction mechanisms. As one of the most critical environmental factors, blue light regulates developmental processes and responses to various stresses. Phototropins (PHOTs), a class of blue-light receptors, are primarily involved in phototropism, chloroplast movement, leaf flattening, and stomatal opening [12,36,37]. A recent study revealed that AtPHOT2 integrates blue light and low-temperature signals to precisely modulate flowering time in *Arabidopsis* [27]. However, the *PHOT* genes in pepper have not been described. In this study, we identified two *CaPHOT* genes in pepper through bioinformatics analysis and molecular cloning (Table 1; Figure S1), which is in line with the majority of the previous reports, such as *Arabidopsis thaliana* [13], strawberry [24], maize [16], *P. aphrodite* [17], and potato [26]. Multiple sequence alignment further showed that all PHOT proteins from pepper, *Arabidopsis*, tomato, maize, and rice contain two similar LOV (LOV1 and LOV2) domains and an STK domain (Figure S2), confirming the reliability of our identification and indicating structural conservation across plant species [38].

To further investigate the functional characteristics of CaPHOTs, we constructed a phylogenetic tree using PHOT protein sequences from pepper, *Arabidopsis*, tomato, maize, and rice. The analysis revealed that pepper PHOTs share closer phylogenetic relationships with those from dicots (*Arabidopsis* and tomato) than with monocots (rice and maize) (Figure 1A), suggesting potential functional conservation between pepper PHOTs and their dicot homologs. Furthermore, nearly all *PHOT* genes exhibited highly similar exon–intron organizational patterns (Figure 1B), indicating a relatively slow evolutionary rate of PHOT genes and implying functional conservation across different plant species. Additionally, both CaPHOT1 and CaPHOT2 were localized to the plasma membrane (Figure 5), consistent with their identity as blue light-activated kinases that typically form complexes with signaling factors at the plasma membrane [39]. Collectively, the conserved plasma membrane localization, highly similar exon–intron structures, and close phylogenetic relationships underscore the strong evolutionary conservation of *PHOT* genes in plants.

Gene expression patterns offer critical insights into gene function. Previous studies have established that *PHOT* genes display distinct tissue-specific expression profiles. For instance, *OsPHOT* transcripts were markedly more abundant in mature leaves than in other rice tissues [15]. In *P. aphrodite*, both *PaPHOT1* and *PaPHOT2* were highly expressed in the labellum, with *PaPHOT1* showing preferential expression in young tissues [17]. In our study, *CaPHOT* genes exhibited peak expression levels in leaves (Figure 2), which are directly exposed to sunlight and harbor a high chloroplast density. This phenomenon aligns with the well-documented roles of *PHOTs* in regulating hypocotyl phototropism, stomatal opening, chloroplast movement, and leaf expansion [11,38,40]. However, under treatments with JA and SA, as well as upon *P. capsici* infection, the expression levels of *CaPHOT1* and *CaPHOT2* displayed significant divergence (Figure 3 and Figure 4), indicating potential functional specialization between these two genes. Similar results were also reported in other plants. For example, *Arabidopsis* PHOT proteins are localized to the plasma membrane, but blue light induces the translocation of a portion of PHOT1 to the cytoplasm and a portion of PHOT2 to the Golgi apparatus and the outer membrane of chloroplasts [39,41,42,43]. Studies have revealed that PHOT1 serves as the primary photoreceptor for phototropism under low-intensity blue light, whereas PHOT2 mediates the chloroplast avoidance response under high-intensity blue light [44,45]. Stphot1 and Stphot2 are non-redundant susceptibility factors in promoting susceptibility to *P. infestans* infection, but only Stphot1 is specifically required for the StNRL1- and blue-light-dependent degradation of StSWAP70 [26].

The distinct spatial expression patterns of *CaPHOT1* and *CaPHOT2* during PCI and hormone treatments suggest that *CaPHOT1* may play a more critical role than *CaPHOT2* in response to PCI in pepper. Therefore, we transiently overexpressed *CaPHOT1* in pepper leaves and evaluated its effect on disease resistance. Under UV light and by trypan blue staining, leaves overexpressing *CaPHOT1* exhibited significantly larger lesion areas compared to the control (Figure 6), confirming that *CaPHOT1* acts as a negative regulator of pepper resistance against PCI. Notably, transient overexpression of *Stphot1* or *Stphot2* enhanced *P. infestans* colonization, while silencing of endogenous *Nbphot1* or *Nbphot2* reduced infection, indicating that both Stphot1 and Stphot2 function as non-redundant susceptibility factors [26]. It is well established that JA and SA are critical phytohormones mediating plant defense responses against various stresses, including PCI [46,47,48]. The induction of resistance-related genes by these hormones is well characterized, with key members playing key roles in plant defense to PCI through JA- or/and SA-dependent pathways. For instance, *CaSBP11* negatively regulates pepper resistance against *P. capsici* by suppressing defense-related gene expression and modulating SA- and JA-mediated signaling pathways [49]. The finding that *CaNHL4* overexpression enhances resistance to *P. capsici*, whereas its silencing increases susceptibility, demonstrates that this gene confers disease resistance by modulating JA/SA-responsive gene expression [46]. In this study, the expression of *CaPHOT1* was up-regulated under JA and SA treatments (Figure 3), which was consistent with the previous results in pepper. Hence, it can be inferred that *CaPHOT1* play a role in pepper against PCI possibly via JA and SA signaling pathways.

## 5. Conclusions

In this study, we molecularly cloned and characterized *PHOT* genes in pepper, analyzing their phylogenetic relationships, conserved domains, and gene structures. Furthermore, we examined the expression profiles of *CaPHOT* genes across various tissues and in response to JA, SA, and *P. capsici* infection (PCI) using RNA-seq and qRT-PCR. Furthermore, the negative role of *CaPHOT1* during PCI was functionally investigated through transient expression assay in pepper plants. Our findings establish a foundation for elucidating the roles of *CaPHOT* genes in pepper growth, development, and stress responses. Furthermore, this study provides a theoretical basis for breeding pepper cultivars tolerant to PCI through gene editing techniques, highlighting the broader potential of *PHOT* gene applications in modern agriculture.

## Figures and Tables

**Table 1:** Identification and characterization of the *CaPHOT* genes in pepper.

Gene Name	Gene ID	Genomic Position	Protein Physicochemical Characteristics				CDS (bp)	gDNA (bp)	Subcellular Localization Prediction
			Length (aa)	MW (kDa)	pI	GRAVY			
*CaPHOT1*	Capana11g000681	Chr11:26818545-26836839	1025	115.39	6.48	−0.706	3078	18,295	Plasma membrane
*CaPHOT2*	Capana08g001309	Chr08:128603069-128615643	960	107.20	8.09	−0.538	2883	12,575	Plasma membrane

## Data Availability

The original contributions presented in this study are included in the article. Further inquiries can be directed to the corresponding authors.

## Supplementary Materials

The following supporting information can be downloaded at: https://www.mdpi.com/article/10.3390/plants14213400/s1. Figure S1. PCR amplification of *CaPHOT1* and *CaPHOT2*. M, DL ladder 5000 DNA Marker; 1, *CaPHOT1*; 2, *CaPHOT2*; Figure S2. Multiple sequence alignments of PHOT proteins from different plant species. Conserved domains of LOV1, LOV2, and STK are underlined. Table S1. The gene-specific primers used in this study; Table S2. PHOTs in representative plant species.
