# Supplementary figures and images for: CaPHOT1 Negatively Regulates the Pepper Resistance to Phytophthora capsici Infection
Source: Plants (Basel). 2025 Nov 6;14(21):3400. doi: 10.3390/plants14213400 (PMC12610167; doi:10.3390/plants14213400)

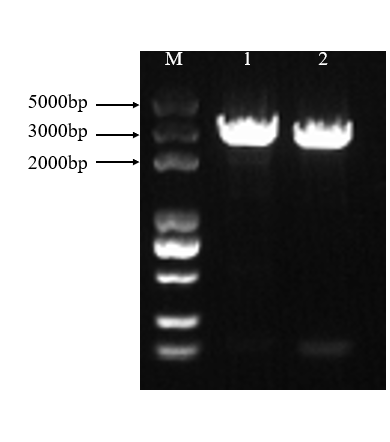

Supplement: Supplementary file 1 [file plants-14-03400-s001.zip › Figure S1.tif]

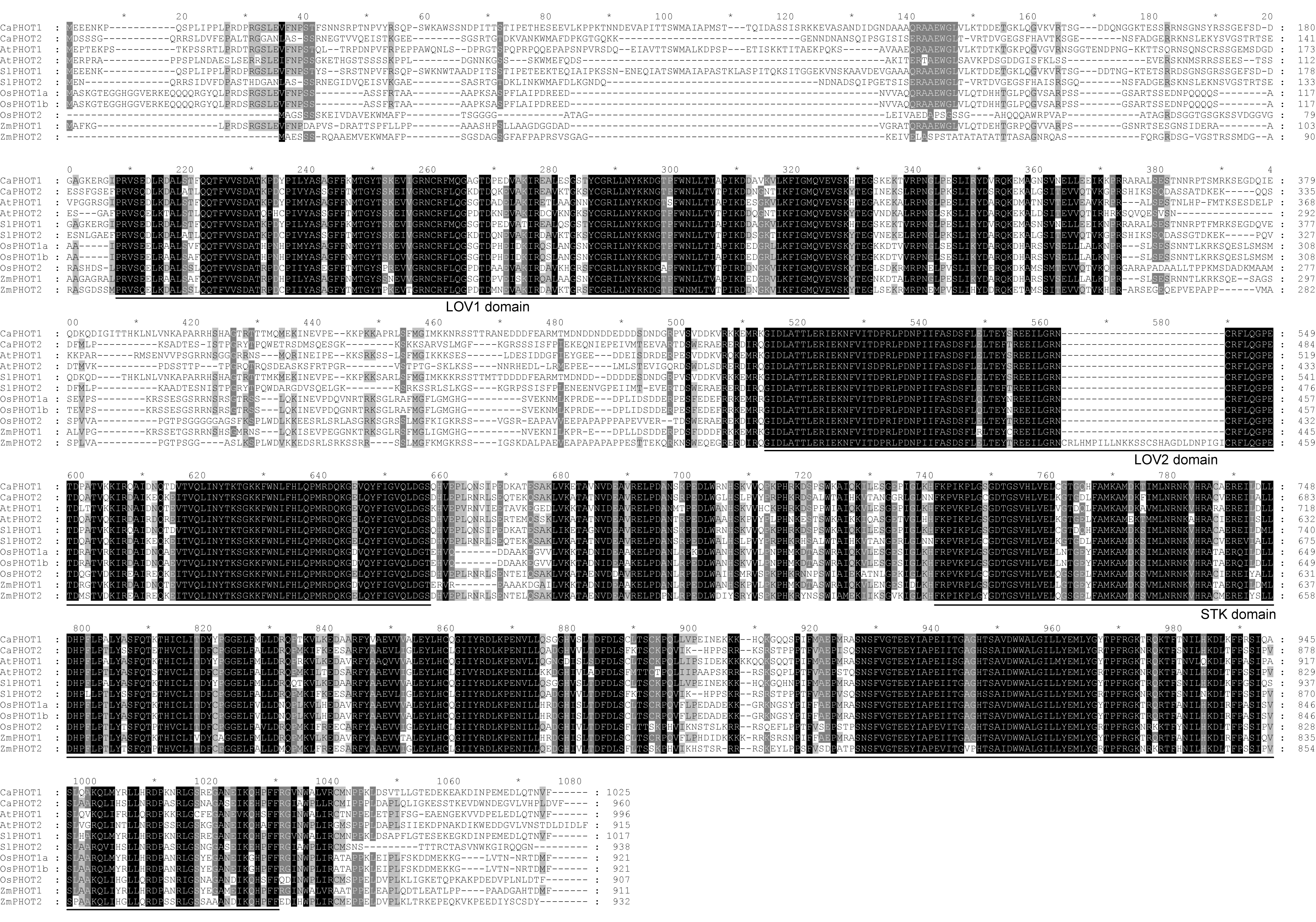

Supplement: Supplementary file 1 [file plants-14-03400-s001.zip › Figure S2.tif]
